# Morphologic transformation of human breast epithelial cells MCF-10A: dependence on an oxidative microenvironment and estrogen/epidermal growth factor receptors

**DOI:** 10.1186/1475-2867-10-30

**Published:** 2010-09-01

**Authors:** Rita Yusuf, Krystyna Frenkel

**Affiliations:** 1 School of Environmental Science and Management, Independent University, Bangladesh, House #27, Road #12, Baridhara, Dhaka-1212, Bangladesh; 2 Department of Environmental Medicine, NYU School of Medicine, PHL Room 802, 550 First Avenue, New York, NY 10016, USA

## Abstract

**Background:**

MCF-10A, immortalized but non-transformed human breast epithelial cells, are widely used in research examining carcinogenesis. The studies presented here were initiated with the observation that MCF-10A cells left in continuous culture for prolonged periods without re-feeding were prone to the development of transformed foci. We hypothesized that the depletion of labile culture components led to the onset of processes culminating in the observed cell transformation. The purpose of this study was to define the factors which promoted transformation of this cell line.

**Results:**

Changes in levels of phenol red (PHR), hydrocortisone (HC), and epidermal growth factor (EGF) with or without estrogen treatment indicated that both oxidative stress- and estrogen receptor alpha (ERα)-mediated pathways contribute to cell transformation. Gene array and Western blotting analyses of cells maintained in our laboratory and of those from other sources documented detectable ERα and ER*beta *(ERβ) in this ERα-negative cataloged cell line. Results also indicate the possibility of a direct association of EGF receptor (EGFR) and ERα in these cells as well as the formation and high induction of a novel ternary complex that includes ERβ (ERα/ERβ/EGFR) in cells grown under conditions facilitating transformation.

**Conclusions:**

Our studies resulted in the development of a growth protocol where the effects of chronic, physiologically relevant alterations in the microenvironment on cellular transformation were examined. From our results, we were able to propose a model of transformation within the MCF-10A cell line in which oxidative stress, ER and EGFR play essential roles. Overall, our work indicates that the immediate microenvironment of cells exerts powerful growth cues which ultimately determine their transformation potential.

## Background

Breast cancer is one of the most common malignancies affecting women in Western countries [1]. Despite extensive research efforts worldwide at understanding and eradicating breast cancer, the cellular processes that lead to the onset of mammary carcinogenesis have yet to be definitively elucidated. Oxidative stress has come under increasing scrutiny in recent years as a causative factor in mammary carcinogenesis. Chronic infection and inflammation, which lead to reactive oxygen species (ROS) generation, are recognized risk factors for cancer development [2]. 17β-Estradiol (E2) [3-6] and epidermal growth factor (EGF) [7,8], two agents that can increase intracellular oxidative stress, are also strongly linked to the development of breast cancer. E2 binding to estrogen receptor (ER) [9-11] and EGF's known properties as a growth factor, [1,12] as well as its putative role in modulating ER expression [13,14], could also lead to cell transformation through the induction of cellular proliferative responses.

Epidemiological evidence and the recognized risk factors implicate estrogens as important etiological agents in the development of breast cancer [9,15-20]. The exact mechanism(s) by which estrogen contributes to the development of breast cancer has not yet been elucidated. Most studies to date have focused on estrogen's role as a promoter of carcinogenesis based on its proven mitogenic activity in cells [9,10,21]. Receptor-based increases in cell proliferation due to estrogen binding are thought to act by either increasing spontaneous errors that make target tissues more susceptible to initiation or enhancing the replication of clones of already initiated target cells [10]. Increasingly, however, the notion that estrogen can function as an initiator of breast cancer *via *ROS generation and consequent oxidative DNA damage is gaining experimental support [3-5,21-24].

Over two decades ago, J. Liehr and coworkers elegantly demonstrated that while 17β-estradiol (E2) exposure induces renal clear-cell carcinoma in Syrian hamsters, 2-fluoroestradiol (2-Fl-E2), a fluorinated estrogen analog that is a potent estrogen but displays reduced metabolic conversion to catechol estrogen metabolites, was non-carcinogenic in this system [25,26]. Oxidation of cytochrome P450-catalyzed catechol estrogen (CE) metabolites, particularly 4-hydroxyestradiol (4-OH-E2), to semiquinones and quinones and their redox cycling, is thought to generate free radicals which can effect oxidative DNA damage [22,23,27,28] leading to mutations and carcinogenesis. 4-OH-E2 is the predominant catechol formed in human mammary fibroadenomas and adenocarcinomas tested [29]. The localized occurrence of a specific estrogen 4-hydroxylase (CYP1B1) in human breast cancer cells, uterine myoma, and rodent target organs of estrogen-induced carcinogenesis has also been observed [29]. Further, formation of 8-hydroxy-2'-deoxyguanosin (8-OHdG) was higher in ERα-positive cultured human breast cancer cells and tissues in comparison to ERα-negative cells [30]. Studies conducted with human sperm and lymphocytes provided evidence that exposure to various estrogenic compounds can lead to free radical-mediated damage as well. This damage was diminished in nearly all cases by catalase, indicating that estrogen-mediated effects act *via *hydrogen peroxide (H_2_O_2_) production [31].

ERα levels can be modulated by EGF [13,14], which was shown to increase oxidative DNA damage in mammary tumor cells coincident with increased malignancy [7]. EGF, a growth factor regulating the proliferation and differentiation of human mammary epithelial cells, is thought to be involved in the pathophysiology of breast cancer [1,12]. Underscoring its significance in mammary carcinogenesis, EGF is present in several human breast cancer cell lines and in 15-30% of human primary invasive breast carcinomas; its mRNA is elevated in ERα-positive human breast cancer cell lines and tumors, and its expression correlates with poor prognosis in breast cancer patients [1]. EGF by itself can increase H_2_O_2 _levels [7,8] and, thus, may be a critical factor in oxidative stress-induced breast cancer.

The culture medium of MCF-10A cells is usually supplemented with various factors such as hydrocortisone (HC), EGF, and phenol red (PHR, a pH indicator), which can affect redox state as well as ER activity. We observed in this study that MCF-10A cells left in continuous culture for prolonged periods without re-feeding were prone to the development of morphologically transformed foci. Our hypothesis was that the depletion of labile culture components induced oxidative stress and led to the onset of spontaneous transformation. However, deliberate manipulation of culture components and treatment with redox active and inactive estrogens indicated both oxidative stress- and ERα-mediated pathways to be operative in the spontaneous transformation of these cells. While MCF-10A cells are characterized as ERα-negative, gene array and western blotting analyses of cells maintained in our laboratory as well as of those obtained from a variety of different sources provided documentation of detectable ERα and ER*beta *(ERβ) in this cell line. Western blotting analysis also indicated for the first time the possibility of a direct association of epidermal growth factor receptor (EGFR) and ERα in the MCF-10A cell line as well as the formation and high induction of a novel ternary complex that includes ERβ (ERα/ERβ/EGFR) in MCF-10A cells grown under conditions facilitating their transformation.

## Materials and methods

### A. Cells and Materials

MCF-10A cells were purchased from American Type Culture Collection (ATCC; Manassas, VA). MCF-10A cells were also kindly provided by Drs. J.D. Yager (Department of Environmental Health Science, The Johns Hopkins University Bloomberg School of Public Health, Baltimore, MD; Source #1), K. Eckert (Gittlen Cancer Research Institute, Penn State College of Medicine, Hershey, PA; Source #2), M. Planas-Silva (Department of Pharmacology, Penn State College of Medicine, Hershey, PA; Source #3), and M. F. Verderame (Department of Medicine, Penn State College of Medicine, Hershey, PA; Source #4). The laboratories at Penn State University that kindly provided MCF-10A cells had obtained these cells independently of one another from different sources. A custom formulation of PHR-free Dulbecco's Modified Eagle's Medium/Nutrient F12 (DMEM/F12) cell culture medium D231SA, trypsin (0.25%, 1×), trypsin-ethylenediaminetetraacetic acid (trypsin-EDTA; 0.05% trypsin, 0.53 mM EDTA, 1×), L-glutamine (200 mM, 100×), and antibiotic/antimycotic (100×) solutions were purchased from Atlanta Biologicals (Norcross, GA). Horse serum (HS) was purchased from Invitrogen (Carlsbad, CA). EGF was purchased from R&D Systems (Minneapolis, MN). Protease inhibitor cocktail tablets were obtained from Roche Molecular Biochemicals (Indianapolis, IN). All other reagents were purchased as described in the text or from Sigma Chemical Company (St. Louis, MO).

### B. Cell Culture

MCF-10A cells were maintained in PHR-free DMEM/F12 culture medium unless otherwise specified. Medium was supplemented with NaHCO_3 _(1200 mg/L), CaCl_2 _(1.05 mM), 5% HS, insulin (10 μg/ml), L-glutamine (2 mM), antibiotic/antimycotic mixture (1%), EGF (20 ng/ml), HC (500 ng/ml), and cholera toxin (100 ng/ml). Cells were fed twice a week and grown to confluence before subculturing. Briefly, cells were washed once with Dulbecco's Phosphate Buffered Saline (D-PBS) and exposed to trypsin for 15-20 minutes before the action of trypsin was stopped with 20% HS-supplemented medium. Cells were then centrifuged at 100 × *g *in a tabletop centrifuge for 5 min and the cell pellet was resuspended in medium and transferred to other flasks. All cells were grown in a single chamber water-jacketed humidified incubator and maintained in a 37°C, 5% carbon dioxide (CO_2_) atmosphere. The number of passages cells have been propagated in a particular type of medium is indicated in parentheses next to the description of the medium [(i.e. -HC/-EGF (#10)]

### C. Assay for Morphologic Transformation

MCF-10A cells maintained in PHR-free 5% HS-supplemented, HC and EGF-containing [+HC/+EGF (+/+)] DMEM/F12 medium were subsequently grown for the 5-week morphologic transformation assay in +/+, -HC/-EGF (-/-), -HC/+EGF (-/+), or +HC/-EGF (+/-) DMEM/F12 media supplemented with 0.5% HS and 240 μg bovine serum albumin (BSA)/ml in the absence or presence of PHR. Cells in these eight medium groups were non-treated (NT) or treated with 0.01% ethanol (EtOH) alone or with 0.01% EtOH solution of 1 nM E2 or 1 nM 2-fluorestradiol (2-Fl-E2). Initially, cells were either left untreated or treated with appropriate agents and then plated in triplicate in 6-well plates at a density of 5 × 10^5 ^cells/well. Thereafter, cells were maintained in continuous culture for 6 weeks, refed and re-treated once a week, and examined microscopically each week for signs of contact-uninhibited growth and the appearance of morphologically transformed foci. Transformed foci were counted once a week from 1-5 weeks at 4× magnification as they appeared along two perpendicular lines intersecting in the center of each well. To assess the reversibility of phenotypic cell alterations, after five weeks, PHR, HC, and EGF were added back singly or together, to cultures that were lacking these factors, and the number of transformed foci was again determined at week 6. The assay was performed once with duplicates of each treatment analyzed. Some treated cells were plated in poly-D-lysine-coated tissue-culture plates in an attempt to increase detailed microscopic visualization and examination of foci.

### D. Western Immunoblotting Analysis

Cells used for Western blot analysis included MCF-10A cells (non-treated and treated under various treatment protocols and media conditions) as well as MCF-10A cells acquired from different laboratories and grown in -PHR, 5% HS, +/+ medium. Total cell extracts were obtained by first trypsinizing and pelleting cells as described in section B of Materials and Methods and washing once with D-PBS. Cell lysis buffer [5.0 M EDTA, 150 mM NaCl, 50 mM Tris HCl, 1% Triton X-100, 1% SDS, 50 mM dithiothreitol (DTT), and protease inhibitor cocktail tablets (1 tablet per 10 ml buffer)] was added to each tube at 100 μl buffer per 1 × 10^6 ^cells and mixed well to lyse the cells completely. Lysates were transferred to microcentrifuge tubes, incubated on ice for 10-30 min., and centrifuged at 12,000 × *g *in a microcentrifuge at 4°C for 15 min. The supernatants were collected and stored at -80°C for subsequent analyses. Alternately, Pierce (Rockford, IL) NE-PER Nuclear and Cytoplasmic Extraction Reagents were used as per the manufacturer's protocol for the stepwise separation and preparation of cytoplasmic and nuclear extracts. Protein content was measured using Bradford Reagent. Proteins (25-30 μg) were resolved by SDS-PAGE in 12% SDS-Tris-HCl polyacrylamide mini-running gels and transferred onto nitrocellulose membranes (BioRad Laboratories; Hercules, CA). Membranes were incubated with primary antibodies to ERα, ERβ, or EGFR at a dilution of 1:1000 in 5% non-fat dry milk-Tris Buffered Saline/Tween (TBS/T) buffer at 4°C overnight, followed by incubation with both the appropriate horseradish peroxidase (HRP)-conjugated secondary antibody at a dilution of 1:10,000 and anti-biotin antibody at 1:1000 dilution in 5% non-fat dry milk in TBS/T at room temperature (RT) for 1 h. Protein was detected using the Western Lightning Chemiluminescent Reagent Plus Kit from PerkinElmer (Wellesley, MA) as per the manufacturer's directions. Antibodies (Ab) and controls used were: **ERα (62A3) **mouse monoclonal Ab, **EGFR **rabbit polyclonal Ab, and anti-biotin Ab (Cell Signaling Technology; Beverly, MA); **ERβ (PA1-313) **rabbit Ab, human, recombinant **ERα RP-310 **and **ERβ (long form) RP-312 **(Affinity BioReagents; Golden, CO); **EGF-stimulated A431 cell lysate **(Upstate Biotechnology; Lake Placid, NY). Peroxidase-conjugated Immunopure goat anti-mouse and sheep anti-rabbit IgG's were used as **secondary antibodies **(Pierce Chemical Company, Rockford, IL)

### E. Gene Expression Analysis of the Human Toxicity/Stress and Estrogen Signaling Pathways

Cells were trypsinized and pelleted according to the protocol outlined in Section B, and RNA was isolated from cells using the RNAqueous RNA isolation system (Ambion, Inc.; Austin, TX) according to the manufacturer's protocol. Immediately afterwards, contaminating DNA was removed using Ambion's "DNA-free" DNase Treatment and Removal Reagents again as per the manufacturer's directions. The RNA supernatants were transferred to new RNAse-free tubes and stored at -80°C. Prior to use in gene expression studies, the concentration and purity of RNA was determined by aliquoting a small amount of the samples in HPLC-grade, RNase-free dH_2_O and measuring absorbance at 260 nm and 280 nm. RNA concentration was calculated using a value of 1A_260 nm _= 40 μg RNA/ml. and its purity assessed by confirming that the ratio of A_260_/A_280 _was near 2.0. Nonrad-GEArray Kit Pathway Specific Gene Expression Profiling System (SuperArray, Inc; Bethesda, MD) was used for the analysis of gene expression after RNA isolation. The detailed manufacturer's protocol was followed for analysis. Briefly, biotinylated cDNA probes were synthesized from 5-10 μg total RNA by reverse transcription using a PCR thermal cycler and SuperArray reagents. Afterwards, cDNA probes were hybridized using a mini hybridization incubator kit reagents to pathway-specific gene expression array membranes (either human toxicity/stress or estrogen signaling) provided by the manufacturer. Finally, membranes were incubated with alkaline phosphatase (AP)-streptavidin, and chemiluminescent detection was performed with the provided CDP-Star substrate and immediate exposure to x-ray film between 0-5 min. Signal intensities were quantitated (semi-log) using UN-SCAN-IT digitizing software (Silk Scientific; Orem, UT) after the x-ray films were scanned onto a computer. Sample signal intensities were normalized against a housekeeping gene's signal intensities. Each membrane contained two spots for each cDNA analyzed. Means of the intensity (in pixels) of the duplicate spots were used for analysis

### F. Statistical Analysis

Significance of differences between two groups was assessed using one-tailed Student's "t" test assuming unequal variances. One-way ANOVA followed by Dunnet's test was utilized to compare all groups to a control group, while One-way ANOVA followed by Tukey's test was used to compare all groups to each other. For all tests, p < 0.05 was considered significant.

## Results

### 1. Simultaneous depletion of HC, EGF, and PHR from culture medium is necessary for the morphologic transformation of MCF-10A cells

**Figure 1:**
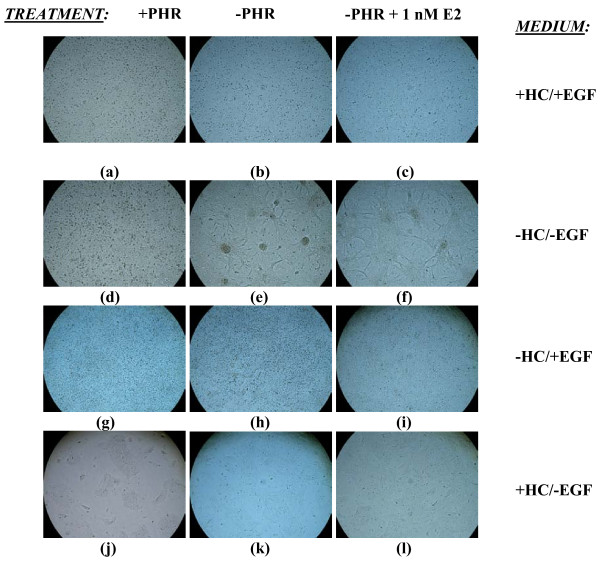
**Effects of PHR, HC, EGF, and E2 on Focus Formation by MCF-10A Cells Cultured for 13 Days**. MCF-10A cells, plated at 5 × 10^5 ^cells/well, were grown in 6-well tissue culture plates under differing culture and treatment (once a week) conditions and photographed at low magnification (phase contrast; 4×) to enable visual indication of the number of transformed foci after13 days in culture. Photographs are representatives of triplicate samples from one experiment.

**Figure 2:**
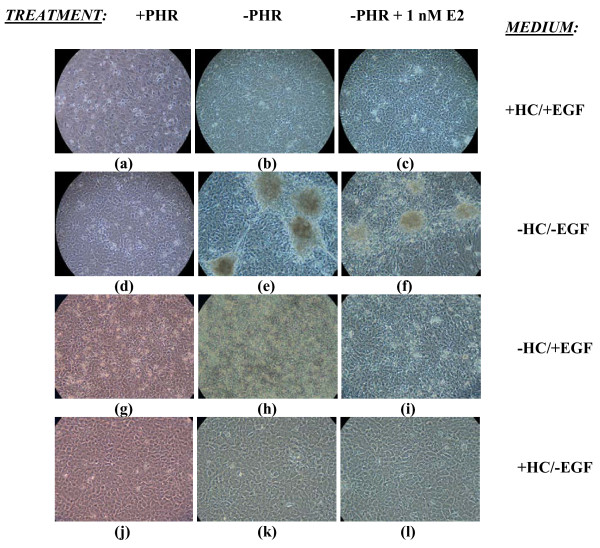
**Effects of PHR, HC, EGF, and E2 on Focus Formation by MCF-10A Cells Grown in Continuous Culture for 5 Weeks**. MCF-10A cells, plated at 5 × 10^5 ^cells/well, were grown in 6-well plates under differing culture and treatment (once a week) conditions and photographed (phase contrast; 10×) after 5 weeks in continuous culture. Photographs are representatives of triplicate samples from one experiment.

**Figure 3:**
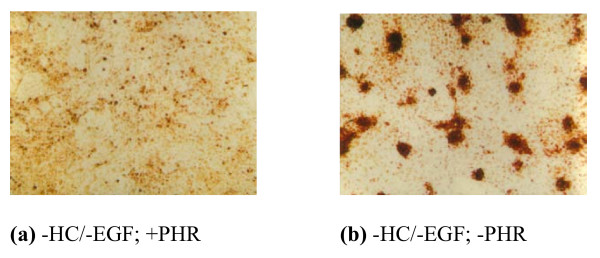
**Effect of PHR Presence in -HC/-EGF Medium on the Appearance of MCF-10A Transformed Foci After 3 Weeks of Continuous Culture**. MCF-10A cells were seeded in triplicate at a density of 6.25 × 10^4 ^cells/well in 6-well tissue culture plates and grown in -HC/-EGF medium either with or without phenol red for 3 weeks in continuous culture. Cells were stained with neutral red (10 mg/ml) and photographed (10× obj.). Photographs are representatives of triplicate wells per culture condition from one experiment.

MCF-10A cells were grown for 5 weeks in continuous culture in 6-well tissue culture plates in PHR-containing or PHR-free medium lacking either one, both, or none of the factors HC and EGF. Cells in these four types of media were also exposed once a week to either 1 nM E2 or 1 nM 2-Fl-E2 in 0.01% EtOH, or just 0.01% ethanol (EtOH) as a control. Within five days in culture, cells grown in the presence of EGF [+HC/+EGF (+/+) and -HC/+EGF (-/+)] became confluent, whereas those grown in the absence of EGF [-HC/-EGF (-/-) and +HC/-EGF (+/-)] displayed a slower growth rate. Cells in the +/- medium exhibited the lowest proliferative capacity. Transformed-looking foci were noted to first appear after 13 days in culture and only in PHR-free -/- cultures (Fig. 1e-f). After 5 weeks, large, prominent foci became apparent but again, only in PHR-free -/- cultures (Fig. 2e-f). The average number of foci per well of a 6-well tissue culture plate was significantly elevated to 79.5+6.50 (p < 0.0005) in -/- medium versus 0 in media containing either HC or EGF (Table 1). An important outcome of these experiments was the observation that the presence of PHR inhibits the appearance of transformed foci (Figs. 1 &2; Table 1). To verify that the piled cells observed in Figs. 1 and 2 were live cells and rule out the possibility of artifactual observation, MCF-10A cells seeded in triplicate at a density of 6.25 × 10^4 ^cells/well in PHR-containing or -deficient -/- medium for 3 weeks in 6-well tissue culture plates were stained with neutral red (10 μg/ml) and photographed. Transformed foci in PHR-deficient -/- medium as well as the monolayer underneath were stained red, confirming the viability of cells both in foci and monolayer (Fig. 3b). The fact that foci were stained deep red while the monolayer was light red suggests piling of cells in foci and proves that foci being observed were not artifacts. Cultures grown in PHR-containing -/- medium did not form foci, as demonstrated by the absence of darkly stained piles of cells (Fig. 3a).

**Table 1:** Effects of PHR, HC, EGF, E2, and 2-Fl-E2 on the Appearance of Transformed Foci in MCF-10A Cells after 5 Weeks in Continuous Culture

Treatments	Number of Foci/Well
(+HC/+EGF) +PHR	0.00

(+HC/+EGF) -PHR	0.00

(+HC/+EGF) -PHR; 1 nM E2	0.00

	

(-HC/-EGF) +PHR	0.00

(-HC/-EGF) -PHR	79.50 +/- 6.50*^, #^

(-HC/-EGF) -PHR; 0.01% EtOH	33.50 +/- 27.50

(-HC/-EGF) -PHR; 1 nM E2	182.00 +/- 2.00**^,Φ^

(-HC/-EGF) -PHR; 1 nM 2-Fl-E2	19.00 +/- 5.00*

	

(-HC/+EGF) +PHR	0.00

(-HC/+EGF) -PHR	0.00

(-HC/+EGF) -PHR; 1 nM E2	0.00

	

(+HC/-EGF) +PHR	0.00

(+HC/-EGF) -PHR	0.00

(+HC/-EGF) -PHR; 1 nM E2	0.00

Effects of PHR, HC, EGF, E2, and 2-Fl-E2 on the appearance of transformed foci in MCF-10A cells after 5 weeks in continuous culture. MCF-10A cells, plated at 5 × 10^5 ^cells/well, were exposed to different culture and treatment conditions for 5 weeks in continuous culture. Cells were maintained and treated once a week in 6-well TC plates. The average number of transformed foci/well that appeared at 5 weeks in triplicate wells of each culture/treatment type from one experiment was used for analysis. Significance of differences was analyzed using One-way ANOVA followed by either Dunnet's or Tukey's test.

Using One-way ANOVA followed by Dunnet's test:

-HC/-EGF/-PHR/E2 vs -HC/-EGF/-PHR/EtOH **p < 0.0005

-HC/-EGF/-PHR vs -HC/-EGF/-PHR/EtOH *p < 0.05

Using One-way ANOVA followed by Tukey's test:

-HC/-EGF/-PHR/E2 vs all groups ^Φ^p < 0.0005

-HC/-EGF/-PHR vs all groups except -HC/-EGF/-PHR/EtOH ^#^p < 0.0005

### 2. β-Estradiol (E2) elevates the morphologic transformation of MCF-10A cells in -HC/-EGF (-/-) medium, whereas 2-Fl-E2 does not

**Figure 4:**
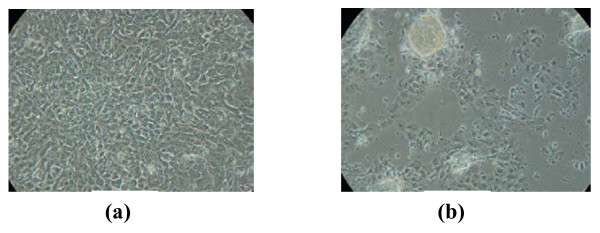
**Effects of 1 nM 2-Fl-E2 Treatment on Focus Formation by MCF-10A Cells Grown in Continuous Culture for 5 Weeks**. MCF-10A cells, plated at 5 × 10^5 ^cells/well, were grown in 6-well TC plates in -PHR/-HC/-EGF medium and treated with 1 nM 2-Fl-E2 once a week. Two different areas of one well were photographed (phase contrast; 10×) in order to show variations in cell morphology and focus formation within the same treatment group at 5 weeks in continuous culture. Photographs are representatives of triplicate samples from one experiment.

In an attempt to correlate the transformative and oxidative capacities of E2 within human breast epithelial cells, the MCF-10A cell line was exposed to 1 nM E2 within the four media types already discussed (+HC/+EGF, -HC/-EGF, -HC/+EGF, and +HC/-EGF). In addition, cells cultured in -/- growth medium were also treated with 2-Fl-E2 to assess the transforming potential of a non-redox cycling estrogen. +/+, -/+, and +/- cultures were already shown to be resistant to transformation, since E2 treatment of these cultures did not induce transformation (Figs. 1 &2; Table 1) within the five-week treatment protocol. However, transformed foci appeared in E2-treated -/- cultures starting from 13 days of exposure (Fig. 1f). Although at 13 days, foci arising in E2-treated cells were light and less prominent (Fig. 1f) than in non-treated cultures (Fig 1e), by 5 weeks the foci in E2-treated cultures were much denser and larger in size (Fig. 2) than they were at 13 days. While foci sizes in the non-treated cells were still larger in comparison to the E2-treated cultures (Fig. 2e-f) even at 5 weeks, E2 treatment resulted in more numerous foci (Table 1), by over 2-fold in PHR-free -/- cultures in comparison to non-treated controls (p < 0.0005). In contrast to E2, exposure of cells to 2-Fl-E2 in -/- medium did not lead to the appearance of transformed foci at 13 days (data not shown). However, at 5 weeks, transformed foci did appear in 2-Fl-E2-treated -/- cultures within certain wells and/or areas of wells where cellular degradation was apparent (Fig. 4b). In wells/areas of apparent healthy cell growth in 2-Fl-E2-treated cultures, no foci were discernable (Fig. 4a). In contrast, E2-induced foci in -/- cultures formed uniformly among and within wells irrespective of cellular disintegration (Fig. 2f). Nonetheless, the apparently transformed foci in 2-Fl-E2-treated cultures were counted and the total foci number was used as a reliable comparative indicator of transformation potential among treatments. After five weeks of growth, E2 significantly (p < 0.0005) enhanced the transformation of MCF-10A cells in -/- medium in comparison to controls, whereas 2-Fl-E2 was unable to increase the basal transformation rate, as evidenced by the formation of a lower number of foci than in controls (Table 1). E2-treated cells formed over five times the number of foci as EtOH controls in -/- medium (Table 1).

### 3. Re-introduction of HC, but not PHR or EGF, to -PHR/-HC/-EGF MCF-10A cultures after 5 weeks can partially reverse morphologic transformation

To evaluate whether microenvironmental changes induce irreversible morphologic transformation of MCF-10A cells, PHR, HC, or EGF alone as well as HC and EGF together were re-introduced to -PHR/-HC/-EGF culture medium after the formation of foci in 5 weeks. Microscopic evaluation after 6 weeks revealed no obvious changes aside from indications of slightly smaller foci in cultures where either HC or EGF was added (data not shown). Importantly, only the addition of HC induced a partial (~40%) but significant (p < 0.01) decrease in foci numbers (Table 2). Adding back EGF, by itself or with HC, resulted only in a non significant increase in the number of foci.

**Table 2:** Reversibility of Morphologic Transformation due to Re-addition of PHR, HC, and/or EGF to MCF-10A Cells Grown for 5 Weeks in -PHR/-HC/-EGF Medium

Group	Culture Condition	Mean Number of Foci/Well +/- SE
1	-PHR/-HC/-EGF	153.50 +/- 0.50
	
	Add Back PHR	157.50 +/- 4.50

2	-PHR/-HC/-EGF	125.67 +/- 4.98
	
	Add Back HC	75.33 +/- 6.74*

3	-PHR/-HC/-EGF	79.50 +/- 6.50
	
	Add Back EGF	92.50 +/- 12.50

4	-PHR/-HC/-EGF	145.50 +/- 12.00
	
	Add Back HC & EGF	156.50 +/- 12.50

MCF-10A cells, plated at 5 × 10^5 ^cells/well, were grown in -PHR/-HC/-EGF medium for 5 weeks in 6-well tissue culture plates, at which time cells were re-exposed to depleted agents once for 1 week. Foci were counted at weeks 5 and 6 and the average number of foci/well in 2 wells per re-added agent(s) before and after re-supplementation from one experiment was used to assess reversibility of morphologic transformation. Significance was analyzed using One-way ANOVA followed by Tukey's test.

Add Back HC vs -PHR/-HC/-EGF *p < 0.01

### 4. MCF-10A transformed foci exhibit extensive interconnections while displaying varying morphologies depending on the growth condition

**Figure 5:**
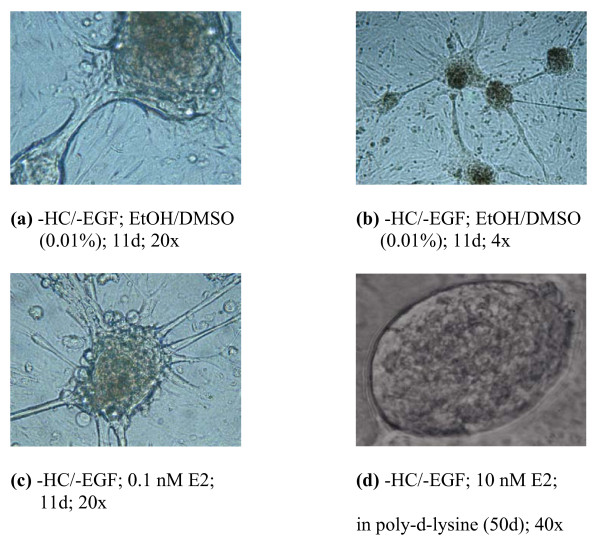
**Morphology of MCF-10A Transformed Foci**. MCF-10A transformed foci, which appear under different culture/treatment conditions, were photographed to document morphologic characteristics. The photograph for panel (d) was taken from a single T25 flask from one experiment. All other photographs are representatives of triplicate wells of 6-well tissue culture plates from one experiment.

**Figure 6:**
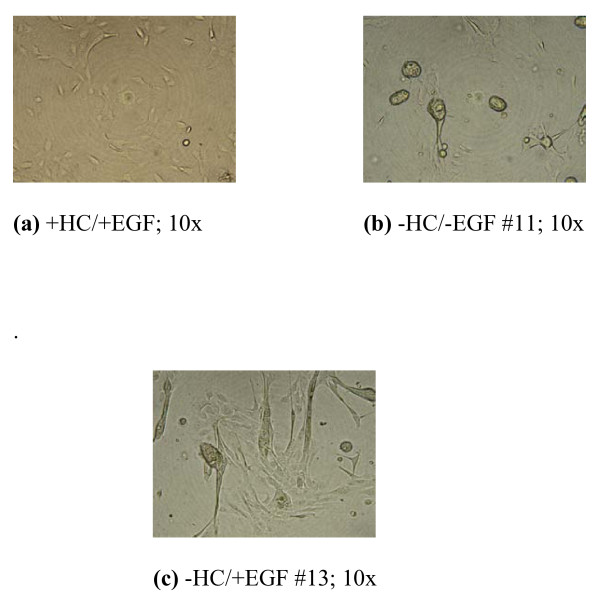
**Effect of HC and/or EGF Withdrawal on MCF-10A Cell Growth and Morphology in PHR-Free Medium after 24 h Culture**. MCF-10A cells were either maintained in fully supplemented medium (+HC/+EGF), grown in HC/EGF-depleted medium (-HC/-EGF) for 11 passages, or grown in HC-deficient medium (-HC/+EGF) for 13 passages. Cells grown in different media types were photographed under phase-contrast microscopy 24 hours after plating. Photographs are representatives of triplicate samples of each culture condition from one experiment.

Slight microenvironmental alterations were seen to profoundly affect the morphology and growth characteristics of MCF-10A human breast epithelial cells. An intriguing characteristic of foci noted in this cell line was the appearance, once foci began to form, of extensive interconnections among them, forming a lattice-like network (Fig. 5a-c). To enable more detailed visualization of transformed foci, 10 nM E2-treated MCF-10A cells were grown in -/- medium until foci formed, trypsinized, and re-plated in poly-d-lysine culture flasks. After 50 days, foci photographed at 40× magnification enabled the identification of a somewhat polarized egg-shaped structure with a distinct, prominent membrane, perhaps of more than one layer, containing the growing contact uninhibited cells within its boundary (Fig. 5d). Growth characteristics of MCF-10A cells grown in the three different types of medium (+/+, -/-, -/+) were examined. Five passages after reaching confluence in their respective medium type, differences among the cells became apparent. Figure 6 shows subconfluent cultures of +/+, -/- (passage #11), and -/+ (passage #13) cells 24 hours after plating. In contrast to +/+ cells, which exhibit normal subconfluent epithelial growth (Fig 6a), -/- cultures already show the presence of small foci growing atop clusters of monolayer cell growth (Fig. 6b). Extensions among foci at this time have already begun to form. MCF-10A -/+ cultures did not form foci at this early time, but displayed a unique morphology of extended, fibroblast-like cells with respect to the other two types of cultures (Fig 6c). Therefore, even a slight microenvironmental alteration can profoundly affect the growth characteristics of human breast epithelial cells, with serious implications regarding their transformation potential.

### 5. MCF-10A cells exhibit detectable levels of both ERα and ERβ gene expression

The necessity for PHR depletion in our studies indicated a need for ERα-mediated events in the transformation of MCF-10A cells. PHR's known weak estrogenicity and binding to ERα [32,33] may suppress receptor's binding of E2, thereby preventing the relatively more potent hormonal and/or oxidative properties of E2 from effecting transformation. Although this cell line is categorized as ERα-negative, our results made it necessary to re-examine the ERα status of these cells. A commercially available gene expression array kit (SuperArray; Bethesda, MD) was utilized for the analysis of human estrogen signaling pathway gene expression in non-treated and 1 nM E2-treated (twice a week for 2 weeks) MCF-10A cells grown under various media conditions. Table 3 demonstrates detectable gene expression of both ERα and ERβ among the different growth and treatment conditions for MCF-10A cells assessed. ERα gene expression was consistently higher in comparison to ERβ expression levels. Although no indicative trends and/or upregulation in ER expression due to varying treatments and culture conditions was noted, the data demonstrate persistent, detectable levels, particularly of ERα gene expression, in this cell line.

**Table 3:** Estrogen Receptor Gene Expression in MCF-10A Cells Under Various Treatment and Culture Conditions

Group	Culture/Treatment Conditions	ERα (Mean Gene Expression)	ERβ (Mean Gene Expression)
1	+HC/+EGF; NT	62.75	9.86
	
	-HC/+EGF (#21); NT	57.98	5.88

2	+HC/+EGF; NT	59.79	15.82
	
	-HC/-EGF (#1); 1 nM E2	57.48	10.21

3	-HC/-EGF (#1); NT	50.77	a
	
	-HC/-EGF (#1); 1 nM E2	53.96	a

ER gene expression in MCF-10A cells maintained in and exposed to various culture/treatment conditions was assessed using a human estrogen signaling pathway-specific gene expression profiling system (SuperArray, Inc; Bethesda, MD) as described in the Methods section. Results shown are those of three different experiments comparing two different culture conditions at a time and using 5-10 μg total RNA. MCF-10A cells grown in culture media either containing or lacking HC/EGF were either left non-treated (NT) or treated twice a week with 1 nM E2 for 2 weeks. Numbers in parentheses indicate the number of passages in that particular medium type. Mean signal intensity (pixels × 10^-4^) values from duplicate spots of both ERα and ERβ per membrane were normalized against those of a housekeeping gene and taken to be representative of mean gene expression.

a) Signal too weak to evaluate

### 6. Western blotting confirms the presence of ERα and ERβ in MCF-10A cells; Possibility of a direct association of ERα and EGFR, with induction of a putative ternary complex composed of ERα/ERβ/EGFR in those cells that are the most prone to transformation

**Figure 7:**
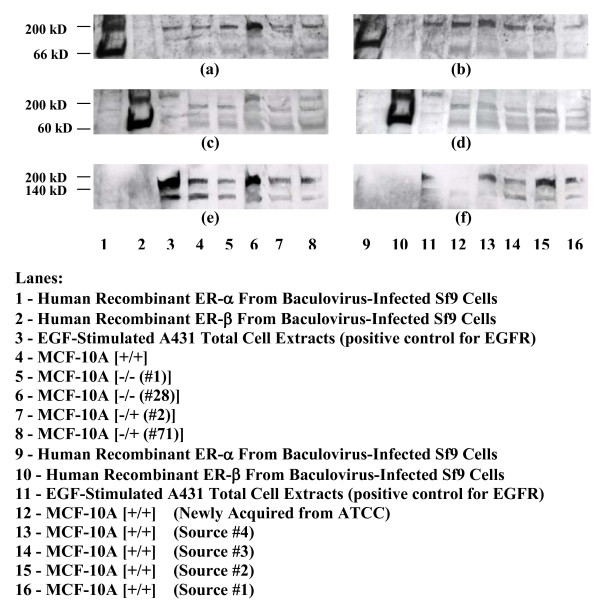
**Analysis of MCF-10A Cells for ERα, ERβ, and EGFR**. Nuclear proteins extracted from MCF-10A cells that were exposed to differing culture conditions were analyzed by Western blotting for the presence of ERα, ERβ, and EGFR. Numbers in parentheses indicate the number of passages in that particular type of medium. Proteins (42 μg/lane) were electrophoresed onto two 12% Tris-HCl gels (lanes 1-8 and 9-16 respectively) and transferred onto nitrocellulose membranes. Membranes were probed with antibodies directed against ERα (a and b), stripped and re-probed with antibodies directed against ERβ (c and d), and stripped a second time and re-probed with antibodies directed against EGFR (e and f). Results shown are those from one experiment.

MCF-10A cells maintained in different growth conditions and those acquired from various laboratories were subjected to Western blotting analysis for ERα, ERβ, and EGFR expression (Fig. 7). Nuclear extracts from 16 samples containing 42 μg protein were electrophoresed onto two 12% SDS-Tris-HCl polyacrylamide gels (gel 1: Fig. 7a, c, e & gel 2: Fig. 7b, d, f) and transferred to nitrocellulose membranes. The two membranes were then probed consecutively with antibodies directed against ERα (Fig. 7a &7b), ERβ (Fig. 7c &7d) and EGFR (Fig. 7e &7f). The first three lanes of both gels contain, respectively, control human recombinant proteins ERα and ERβ from baculovirus-infected Sf9 cells, and total cell lysate from EGF-stimulated A431 cells (positive control for EGFR) (Fig. 7a-f; lanes 1-3 & 9-11). All other lanes contain MCF-10A sample nuclear extracts. Membranes were first probed with antibodies directed against ERα, which detect a 66 kD protein. These blots are depicted in Figure 7a-b and appear to show very light but detectable bands at 66 kD in all MCF-10A sample lanes (Fig. 7a-b; lanes 4-8 & 12-16) corresponding to ERα, as corroborated by staining in this region in the ERα positive control (Fig. 7a-b; lanes 1 & 9). Bands are absent in the lane containing recombinant ERβ protein (Fig. 7a-b; lanes 2 & 10), verifying the absence of non-specific binding. Interestingly, EGFR control cell extracts from A431 cells also appear to indicate the presence of even lighter, but still detectable bands at 66 kD (Fig. 7a-b; lanes 3 & 11). A431 are not known to express ERα, but such a possibility is suggested by observed growth inhibition by the antiestrogen tamoxifen [34] and growth stimulation by the estrogenic compound genistein [35] in A431 cells. The same two membranes were re-probed with antibodies directed against ERβ (Fig. 7c-d). Bands around 60 kD appear to be present in the lane containing the recombinant ERβ (Fig. 7c-d; lanes 2 & 10), which has a molecular weight of 59.2, and in all other MCF-10A samples tested (Fig. 7c-d; lanes 4-8 & 12-16). Bands are not present in the lane containing recombinant ERα (Fig. 7c-d; lanes 1 & 9), again confirming the absence of non-specific antibody binding. A431 control cell extracts do not show the presence of ERβ (Fig. 7c-d, lanes 3 & 11) as they did ERα (Fig. 7a-b; lanes 3 & 11). Re-probing the gels with antibodies directed against EGFR (Fig. 7e-f), which detect the protein at ~175 kD, indicates the presence of bands between 140 and 200 kD, again in all MCF-10A samples (Fig. 7e-f; lanes 4-8 & 12-16) and in the EGF-stimulated A431 cell extracts (Fig. 7e-f; lanes 3 & 11), which serve as a positive control for EGFR. Bands are not seen in lanes containing human recombinant ERα (Fig. 7e-f; lanes 1 & 9) or ERβ (Fig. 7e-f; lanes 2 & 10). These data taken together appear to confirm the presence of ERα, ERβ, and EGFR in all MCF-10A samples tested, even in those obtained from different laboratories, and suggest the possible presence of ERα in the human squamous cell carcinoma line A431. What is even more intriguing in these Western blots is the presence of a band at ~200 kD in all MCF-10A samples and A431 samples that is detected by antibodies directed against both EGFR and ERα (Fig. 7a-b &7e-f; lanes 3-8 & 11-16). This band appears to be induced in MCF-10A cells grown for 28 passages in HC- and EGF-depleted medium (Fig. 7a & e; lane 6). This band is not seen only in the photograph (Fig. 7f, lane 12), but is present as an extremely faint band in the original x-ray film of the newly acquired MCF-10A cells grown in +/+ medium. While highly speculative, an association between EGFR and ERα in both MCF-10A and A431 cells, which is upregulated in MCF-10A -/- #28 cells is one possible explanation for these results. Interestingly, in blots which were analyzed with ERβ antibodies (Fig. 7c-d), this ~200 kD band is apparent only in A431 (Fig. 7c-d; lanes 3 & 11), MCF-10A -/- #28 (Fig. 7c; lane 6), and MCF-10A -/+ #71 (Fig. 7c; lane 8) cells. Again, one possible explanation for this, which will need to be explored further in order to be confirmed, is the existence of a ternary association of ERα/ERβ/EGFR unique to these particular cells. The absence of detectable ERβ at ~60 kD (Fig. 7c-d; lanes 3 & 11) in A431 cells may be an indication of low levels of ERβ in these cells which preferentially associate with EGFR/ERα complex. Overall, however, Western blot analysis appeared to demonstrate detectable levels of ERα, ERβ, and EGFR in MCF-10A cells grown under various culture conditions in our lab and those obtained from a variety of sources. These data also initiate speculation of a direct association of EGFR and ERα in MCF-10A cells. MCF-10A cells grown long-term in HC/EGF-depleted medium (-/- #28) appear to display a high induction of this complex but with the added presence of ERβ. This putative ternary complex is also slightly induced in MCF-10A cells chronically depleted of HC and propagated for a longer time (-/+ #71), presented in lane 8.

### 7. Modulation of ER-responsive genes in MCF-10A cells by E2 and redox manipulation

In support of the role of an active ERα component in MCF-10A cell transformation, Table 4 demonstrates that 1 nM E2 treatment of -/- MCF-10A cultures upregulated prolactin (PRL) gene expression by over 6-fold, which was statistically significant (p< 0.05), while it down-regulated progesterone receptor (PR) gene expression by over 2-fold, suggesting hormonal estrogen responsiveness of this cell line. PRL gene expression was also seen to be modulated in the presence of EGF by the long-term depletion of HC, where -/+ (#21) MCF-10A cells exhibited a decrease in PRL gene expression by 4-fold (p < 0.05) in comparison to +/+ cultures (Table 5). Expression of several other genes involved in the estrogen signaling pathway was also modulated in response to the chronic depletion of HC from MCF-10A culture medium (Table 5). Expression of the genes c-fos and c-jun was diminished by nearly 2- and over 3-fold, respectively, while c-myc expression was abrogated entirely. ER-binding fragment-associated antigen9 (EBAG9) and EGF gene expression were downregulated by over 4-fold each, while H-*ras *by 3-fold.

**Table 4:** Comparison of Progesterone Receptor and Prolactin Gene Expression in 1 nM E2-Treated *versus *Non-treated MCF-10A -HC/-EGF (#1) Cells

Group	Gene	Gene Expression in -HC/-EGF (#1) Cells (Non-Treated)	Gene Expression in -HC/-EGF (#1) Cells (1 nM E2)
1	PR	5.17	2.27

2	PRL	0.82	5.52 *

MCF-10A cells maintained in +HC/+EGF medium were grown for 1 passage in -HC/-EGF medium and either left not treated (NT) or were treated twice a week for 2 weeks with 1 nM E2. Gene expression analysis of genes involved in the human estrogen signaling pathway in MCF-10A cells grown under these two treatment conditions were compared using a gene expression profiling system (SuperArray, Inc; Bethesda, MD) as described in the Methods section. Mean signal intensity (pixels × 10^-4^) values from duplicate spots of each cDNA per membrane were normalized against those of a housekeeping gene and taken to be representative of mean gene expression. Results shown are those of one experiment comparing two different treatment conditions. Significance of differences between expression of PR and PRL genes in MCF-10A -HC/-EGF (1 nM E2) cultures *versus *expression of their respective controls in MCF-10A -HC/-EGF (NT) cultures was analyzed using one-tailed Student's "t" test assuming unequal variances. *p < 0.05

**Table 5:** Comparison of Estrogen Signaling Pathway Gene Expression in MCF-10A **-**HC/+EGF (#21) *versus *+HC/+EGF Cells

Group	Gene	Mean Gene Expression in +HC/+EGF	Mean Gene Expression in -HC/+EGF #21
1	c-fos	22.50 +/- 4.70	12.36 +/- 3.37

2	c-jun	11.87 +/- 2.78	3.83 +/- 2.20

3	c-myc	2.20 +/- 0.03	0.00 +/- 0.00**

4	EBAG9	8.11 +/- 3.2	1.82 +/- 0.79

5	EGF	8.05 +/- 2.98	2.08 +/- 0.25

6	H-***ras***	6.75 +/- 1.35	1.95 +/- 1.85

7	PRL	32.19 +/- 2.42	8.57 +/- 0.16*

8	pS2	1.16 +/- 0.91	3.73 +/- 1.84

MCF-10A cells maintained in +HC/+EGF medium were either kept in this medium or subsequently grown for 21 passages in -HC/+EGF medium. Gene expression analysis of genes involved in the estrogen signaling pathway in MCF-10A cells grown in these two media types were compared using a gene expression profiling system (SuperArray, Inc; Bethesda, MD) as described in the Methods section. Mean signal intensity (Pixels × 10^-4^) values from duplicate spots of each cDNA per membrane were normalized against those of a housekeeping gene and taken to be representative of mean gene expression. Results shown are those of one experiment comparing two different culture conditions. Significance of differences between expression of each gene in MCF-10A -HC/+EGF #21 cultures *versus *expression of its respective control in MCF-10A +HC/+EGF cultures was analyzed using one-tailed Student's "t" test assuming unequal variances. *p < 0.05, **p < 0.005

## Discussion

The mandatory depletion of HC, a potent anti-inflammatory agent thought to decrease oxidative stress in cells, in order to transform cells suggests that an oxidant milieu is critical to the carcinogenic process (Figs. 1 &2). However, re-addition of HC, significantly (p < 0.01), but only partially, reversed the morphologic transformation seen in 5-week -/- MCF-10A continuous cultures (Table 2). The inhibitory effect of added HC on cell transformation and the reversible nature of its action have been documented in various cell types [36-38]. For example, the presence of HC reversibly mediated growth inhibition as well as anchorage-dependence of rat C6 glioma cells and blocked colony formation in agarose [36,37]. HC-mediated ROS suppression [39,40], decrease of nuclear NF-κB [41], and increases in antioxidant enzymes [42] are likely responsible for such transformation-retarding effects.

Transformation of MCF-10A cells, however, was also dependent on the simultaneous depletion of EGF from the culture medium (Figs. 1 &2); hence, EGF withdrawal-mediated ROS generation could play a role in such transformation. In mouse proximal tubular (MPT) cells, EGF deprivation was shown to elevate cellular superoxide anion radical levels and induce apoptosis [43]. However, by itself, EGF can trigger H_2_O_2 _production [7,8] and thus, the finding that its presence inhibits transformation supports the possible outgrowth of EGF-independent clones and suppression of EGFR activity as important events in the transformation pathway [44,45] as well. In fact, adding back EGF, both by itself or with HC resulted in a slight increase in the number of foci (Table 2) and points to the possible outgrowth of EGF-autonomous cells, which then become hypersensitive to the action of EGF perhaps due to the acquisition of a constitutively active EGFR pathway. Lack of EGF in cell culture medium has previously been linked to the spontaneous transformation of HMT-3522 cells [46,47], to carcinogen-initiated neoplastic transformation of Syrian golden hamster pancreatic duct cells [48], and to benzo[a]-pyrene (BP)-enhanced cell proliferation in MCF-10A cells [49].

Our studies showed that transformation rates of MCF-10A cells treated with 1 nM E2 were elevated by over 5-fold in comparison to those of EtOH controls, only within a pre-existing oxidant microenvironment generated by HC and, possibly, EGF depletion (Table 1). The probability that E2-mediated transformation relies on the generation of ROS is indicated by the observation that 1 nM 2-Fl-E2, an estrogen whose metabolism leads to the formation of lower levels of oxidants [4,25-27,50], is incapable of increasing transformation in MCF-10A cells over EtOH controls (Table 1). Studies previously conducted in animals and in various cell models implicate estrogens in transformation, ROS generation, and oxidative DNA damage, particularly 8-OHdG [25,28,30-32,51,52]. Yet, our data also implicated estrogen receptor-mediated effects on cellular transformation. MCF-10A cells exposed to E2 are refractory to transformation even in the absence of HC (-/+ cultures) but in the presence of EGF (Figs. 1 &2; Table 1), implying a need for the possible upregulation of ER-α, due to EGF withdrawal, within the carcinogenic process in this model. Low EGF concentrations in a low serum-containing medium stimulated growth of high ERα-expressing human breast cancer cell lines A431 and BT20, while high EGF doses inhibited their growth [53]. Taken together, the data implicate EGF independence and E2-generated ROS and/or ERα-mediated events as possible contributors to MCF-10A transformation.

The presence of ERα in this ERα-negative categorized cell line and its importance in transformation is underscored by observed transformation suppression in the presence of PHR (Figure 3; Table 1) at 5 days (data not shown), 13 days (Fig. 1), and 5 weeks (Fig. 2). PHR, a known weak estrogen [32,33] used as a pH indicator at a concentration of 15-45 μM in most tissue culture media, can bind to the ERα of MCF-7 human breast cancer cells at an affinity of 0.001% of E2 and was seen to reduce ERα-mediated growth stimulatory processes of exogenous estrogens [32]. The PHR concentration of media used in the present study (21.5 μM) could, therefore, effectively have blocked E2-mediated hormonal and/or oxidative effects on foci formation, as was observed. MCF-10A cells are normally cultured in medium supplemented with horse serum (HS), which contains estradiol. It is possible that chronic exposure of MCF-10A cells to picomolar (~6 × 10^-12 ^M) estradiol contained in HS led to upregulated ERα expression and contributed in part to the transformation of MCF-10A cells seen in -/- medium even in the absence of added E2 (NT and ethanol controls). We found that ERα-mediated events in MCF-10A cell transformation most likely constitute irreversible alterations since re-introducing PHR to culture medium had no effect on the number of foci, once formed (Table 2).

Gene expression arrays confirmed the expression of both ERα and ERβ in MCF-10A cells (Table 3) as well as estrogen responsive genes (Tables 4 &5). The persistent, detectable levels of ERα and ERβ observed among varying culture conditions and treatments (Table 3), even in cells newly purchased from ATCC, provide evidence contradicting the classification of the MCF-10A cell line as ERα-negative. Hormonal estrogen responsiveness was also indicated by the observation that a 1 nM E2 treatment of -/- MCF-10A cultures upregulated prolactin (PRL) gene expression by > 6-fold, while it down-regulated progesterone receptor (PR) gene expression by >2-fold (Table 4). Such modulation has important implications for mammary cell differentiation/proliferation and cancer development. Pituitary prolactin levels are known to be increased due to exposure to exogenous estrogens [54], promote mammary cancer in rats and mice [55] and can activate Ras in rat lymphoma cells [56] with recent studies linking circulating levels to breast cancer [57]. PR, as well, is known to induce mammary epithelial cell proliferation [58,59] and contribute to mammary tumorigenesis [58]. Similar to our findings, suppression of PR gene expression in human breast epithelial cells ML-20 and KPL-1 within a hypoxic microenvironment promoted malignancy [60]. Interestingly, we noted that HC withdrawal was noted to modulate expression of estrogen responsive genes pS2, EBAG9, and PRL and genes involved in estrogen signaling such as EGF, c-fos, c-jun, c-myc, and H-ras (Table 5), which may be the result of an attempt by the cell to combat oxidative stress-induced cellular transformation.

The reasons for down-regulated EGF expression due to HC withdrawal are unclear. However, the presence of EGF inhibited MCF-10A foci formation even when cells were continuously treated with E2 (Table 1). EGF withdrawal was previously documented to transform human breast epithelial cell line HMT-3522, where EGFR suppression was posited to promote estrogen-responsive breast cancer [44,45]. As well, low EGF levels present in low serum-containing medium stimulated growth of human breast cancer cell lines A431 and BT20, expressing high ERα levels, while high EGF concentrations inhibited cell growth [53]. Interestingly, in EGF-depleted MCF-10A cells, increased ROS generation due to benzo[a]pyrene-quinone (BPQ) exposure was seen to activate EGFR [49]. In other studies, redox regulation of ER was also apparent, where H_2_O_2_-induced oxidative stress in MCF-7 and T-47 D human breast cancer cells led to a minimal upregulation of ER-α but a significant increase in ER-β levels [61]. The initial depletion of HC and EGF from the growth medium of MCF-10A cells could lead to the upregulation of ER expression due to both EGF withdrawal-mediated effects and elevated oxidative stress. At the same time, increased oxidant levels concomitant with EGF-withdrawal may also activate EGFR in these cells. EGF hypersensitivity was already noted in our system (Table 2).

Further support for increased ER and EGFR activities due to increased oxidative stress and concomitant EGF withdrawal was provided by the possible existence of a novel, yet still highly speculative, direct association of EGFR and ERα in MCF-10A seen to be induced and believed to include the presence of ERβ in chronic HC/EGF-depleted MCF-10A cells (Fig. 7), which are the most prone to transformation. A puzzling observation in the Western blots showing this ternary complex formation is the presence of the ~200 kD band in lanes containing the recombinant proteins ERα and ERβ synthesized in baculovirus-infected Sf9 cells (Fig. 7a-b; lanes 1 & 9 and Fig. 7c-d; lanes 2 & 10). This observation can only be explained by copurification of these recombinant proteins with contaminating host EGFR proteins. Yet, Sf9 are insect spodoptera frugiperda cells do not contain human EGFR. However, Sf9 cells do contain a growth-blocking peptide receptor (GBPR) having a tyrosine phosphorylation subunit, which can bind human EGF, and can be detected in gels by probing with anti-human EGFR antibody [62]. Thus, association of GBPR with ERα or ERβ during their synthesis in Sf9 cells would explain the presence of the ~200 kD band in bands containing the recombinant proteins and probed with their respective antibodies. Detection of the ~200 kD band in lanes containing both recombinant ERα and ERβ indicate that GBPR can associate with both proteins, yet these bands would not cross-react with both ERα and ERβ antibodies, as seen, since only one protein would be synthesized at a time in Sf9 cells. The absence of a ~200 kD band in lanes containing recombinant ERα or ERβ in gels probed with EGFR antibodies (Fig. 7e-f; lanes 1-2 & 9-10) may be due to the fact that the EGFR moiety detected by the particular EGFR antibody used is not present in GBPR.

Induction of this, as yet speculative, ERα/ERβ/EGFR ternary complex formation may provide an explanation and plausible mechanism for the increased EGF and E2 sensitivity noted in the transformation of this cell line. Chronic withdrawal of HC/EGF from MCF-10A cell cultures seems to strongly facilitate the formation of this putative ERα/ERβ/EGFR ternary complex, a possible manifestation of the ER and EGFR upregulation induced by increased ROS and EGF deficiency in the microenvironment, thereby conferring both EGF and E2 hypersensitivity to cells. While work by other laboratories have implicated either ER or EGFR upregulation/activation due to the actions of EGF withdrawal and increased oxidative stress either by themselves or together, the present study indicates increased activation of both ER and EGFR in the MCF-10A cell line due to the simultaneous effects of both increased oxidant stress and EGF withdrawal. The transformation-enhancing action of such EGF and E2 hypersensitivity can be mediated by the induction of this possible ERα/ERβ/EGFR ternary complex noted to occur under EGF-deficient, pro-oxidant conditions. Marquez *et al*. have also demonstrated a novel direct interaction between ER and EGFR after EGF treatment of MCF-7 cells where EGFR tyrosine kinase phosphorylates ERα at tyrosine-537 and tyrosine-43, possibly leading to estrogen-independent activation of ER-mediated transcription and cell proliferation [63,64]. Others have reported similar results [65]. Proteins recognized by ER-α and ER-β monoclonal antibodies were found in close association to EGFR in lung tumor cells [66]. As well, estrogen was seen to promote an association between extranuclear ER-α and the EGFR family member ERBB4 in the T47 D breast cancer cell line [67]. Such cross-talk can activate diverse downstream signal transduction pathways which regulate cell proliferation [66,68]. In addition, bi-directional cross talk between ER and EGFR can enhance the individual actions of steroids [69]. Thus, augmented cell proliferation and survival responses [14,63,70-72] due to ER/EGFR interactions in MCF-10A cells can possibly lead to their transformation. Several laboratories have posited the probable co-existence and/or necessity for ER-mediated proliferative effects and CE-mediated genotoxic and oxidative events in carcinogenic process [20,73,74]. Results from the present study indicate this to be the case in the transformation of MCF-10A cells.

## Conclusions

**Figure 8:**
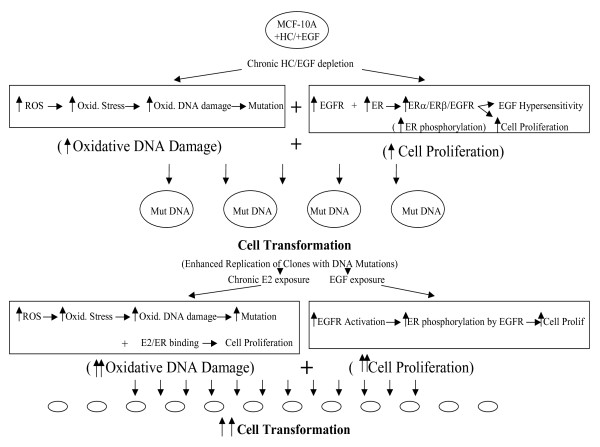
**Proposed Model for the Transformation of Human Breast Epithelial Cells MCF-10A**.

A model for the transformation of human breast epithelial cells MCF-10A (Fig. 8) is proposed where initial chronic HC/EGF-deprivation increases ROS formation leading to elevated oxidative stress and resultant oxidative DNA damage-induced gene mutations. At the same time, the depletion of HC and EGF induce ER upregulation along with activation of EGFR leading to upregulation of the postulated ERα/ERβ/EGFR complex. This, in turn, leads to enhanced ER phosphorylation by EGFR as well as increased sensitivity to the effects of endogenous EGF, both of which induce cell survival and/or proliferative pathways. Consequently, elevated oxidative stress concomitant with increased cell proliferation amplifies replication of clones carrying DNA mutations. Further chronic exposure of these initiated cells to E2 can again cause increased CE-mediated ROS generation and oxidative DNA damage-induced mutations. The prior upregulation of ER and ER phosphorylation also enhances E2/ER binding leading to increased cell proliferation. Simultaneous exposure of initiated cells to EGF also augments EGFR activation and ER phosphorylation, again resulting in increased cell proliferation. More pronounced oxidative DNA damage and cell proliferation at this second stage, where exogenous E2 and EGF act, significantly raise cell transformation rates, once again due to the increased replication of clones with DNA mutations.

Part of the cellular machinery enabling such transformation is suggested by the unique cellular interconnections we observed within cultures grown in media conducive to transformation (Figs. 5 &6). While the exact function of these interconnections is unknown, it is possible that they facilitate the channeling and/or sharing of nutrients, growth factors, etc., needed for foci survival. The discovery of the *de novo *creation of actin-based tunneling nanotubules (TNTs) which arise due to medium deprivation and are capable of transporting organelles between cells in different cells [75] provides support for this type intercellular communication.

This study demonstrates that microenvironmental manipulations, namely the simultaneous depletion of HC and EGF from culture medium, which increase intracellular oxidative stress, can induce transformation in the MCF-10A cell line. We have developed a growth protocol in which the effect of chronic, physiologically relevant microenvironmental alterations on cellular transformation can be examined. Each analysis of duplicate or triplicate samples from single experiments presented in this study represents the culmination of extensive prior work in the selection of optimal culture and treatment conditions. Exposure of cells to chronic, physiologic doses of E2 were required to effect transformation, conditions that mimic the lifetime exposure of the human breast to endogenous estrogens which is believed to play a part in the onset of breast cancer [76,77]. Both E2-mediated oxidative effects and ER-mediated events were found necessary to effect transformation. Our work provides the first indications suggesting a direct association of EGFR and ERα as well as a possible ternary association (ERα/ERβ/EGFR), which is highly induced in chronically HC/EGF-depleted MCF-10A cells which are the most prone to transformation. Overall, results indicate that the immediate microenvironment of cells exerts powerful growth cues which ultimately determine their transformation potential.

## Competing interests

The authors declare that they have no competing interests.

## Authors' contributions

RY conceptualized the study, designed and carried out all experiments, analyzed the data, carried out the statistical analyses, and drafted the manuscript. KF provided substantial intellectual input into the conceptualization and design of the study, interpretation of the data, and revision of the manuscript for final submission. All authors have read and approved the final manuscript.
